# Excited Catatonia in Autism Spectrum Disorder: A Case Series

**DOI:** 10.3389/fpsyt.2021.674335

**Published:** 2021-05-11

**Authors:** Nora Kathleen Burns, Kathleen Grissett, Marc Macaluso, Mohsin Raza, Barbara Gracious

**Affiliations:** GME Psychiatry, Orange Park Medical Center, Orange Park, FL, United States

**Keywords:** catatonia, ECT, autism spectrum disorder, excited catatonia, pediatric

## Abstract

**Introduction:** Autistic catatonia is an under-recognized debilitating syndrome with long-lasting negative effects for families, healthcare workers, and high-cost to the healthcare system. In this report, we describe two cases of excited catatonia in young men diagnosed with autism. Both endured a delay to diagnosis and difficulty to obtain appropriate treatment.

**Main concern:** Each patient had a change in behavior from their baseline but with differences in severity and onset. The diagnosis in the first patient was made after only 3 months as the change was dramatic and sudden. Yet, despite a confirmed diagnosis, it was difficult to treat as the importance of M-ECT was not recognized by the clinicians. The second patient had been suffering for more than 5 years with a slow progression of worsening aggressive symptoms. The aggression was so uncontrollable that the patient required sedation, intubation and daily ECT. Both suffered from agitation, unprovoked aggression, urinary incontinence, stereotypic, and OCD behaviors and compulsive masturbation.

**Primary Diagnosis, intervention/outcomes:** Both patients were diagnosed with autism, one high-functioning, attending high school and working a part-time job, the second low-functioning, nearly non-verbal, isolated to home and ABA school. The first patient's diagnosis of catatonia was only suspected after five psychiatric admissions and more than 20 medication trials. Lorazepam challenge was effective, he was treated with a short series of ECT but each time the treatments were tapered, the aggression returned. Ultimately, stabilized on weekly ECT. The second patient's behavior was escalating over a 5 month period, to the point, the aggression was uncontrollable. He presented to the ED under involuntary hold and the behavior could not be stabilized to the point that emergent ECT was initiated.

**Conclusion:** Two cases of autistic catatonia diagnosed and treated within a year time span at a small community hospital indicates that this diagnosis is more common than previously recognized. We propose screening all patients with neurodevelopmental disorders with the Bush-Francis and Kanner scales to diagnose and treat them appropriately.

## Background

Autistic catatonia, an underrecognized debilitating syndrome, creates long-lasting adversity for patients, families, and healthcare workers, with high cost burdens to healthcare systems. We discuss the presentation, diagnosis, and treatment of two transitional-age patients with autistic catatonia. Patient (A), an 18-year-old male with high-functioning autism and attention deficit hyperactivity disorder (ADHD), presented with repetitive behaviors, mutism, disinhibition, and periods of increased psychomotor agitation and aggression. Patient (B), a 17-year-old male with autism and profound intellectual disabilities, presented with increased aggression, urinary incontinence, disinhibited behavior, posturing, grimacing, and running attacks. Both endured delays in diagnosis of catatonia and challenges obtaining appropriate treatment, largely due to their autism. Both eventually underwent electroconvulsive therapy (ECT) with significant improvement and both required maintenance treatment (M-ECT).

Although still not well-understood or easily diagnosed, descriptions of autistic catatonia date back more than 20 years (1, 2). Recognizing a new case is difficult, as presentations are heterogeneous, with fluctuations in severity and features of both autism and catatonia (3–5). To increase timely detection and treatment in these vulnerable patients, we aim to improve awareness of clinical presentations of autistic catatonia, prompt routine use of diagnostic screening instruments, and educate on appropriate neurobiologic treatments.

## Case A

Mr. A, an 18 year-old high school junior with high-functioning autism, was an athlete in Special Olympics, worked as a grocery store stocker, and had aspirations of studying sharks in a university program for students with autism. After 1 week of insomnia, he presented to the Emergency Department (ED) with delusions and aggression including head-banging. His mother reported a stressful incident 2 weeks prior, when Mr. A awoke to a SWAT team evacuating his building for an active shooter situation. His entire family was rushed out of their apartment to await the “all clear” outside for several hours.

**Figure 1:**
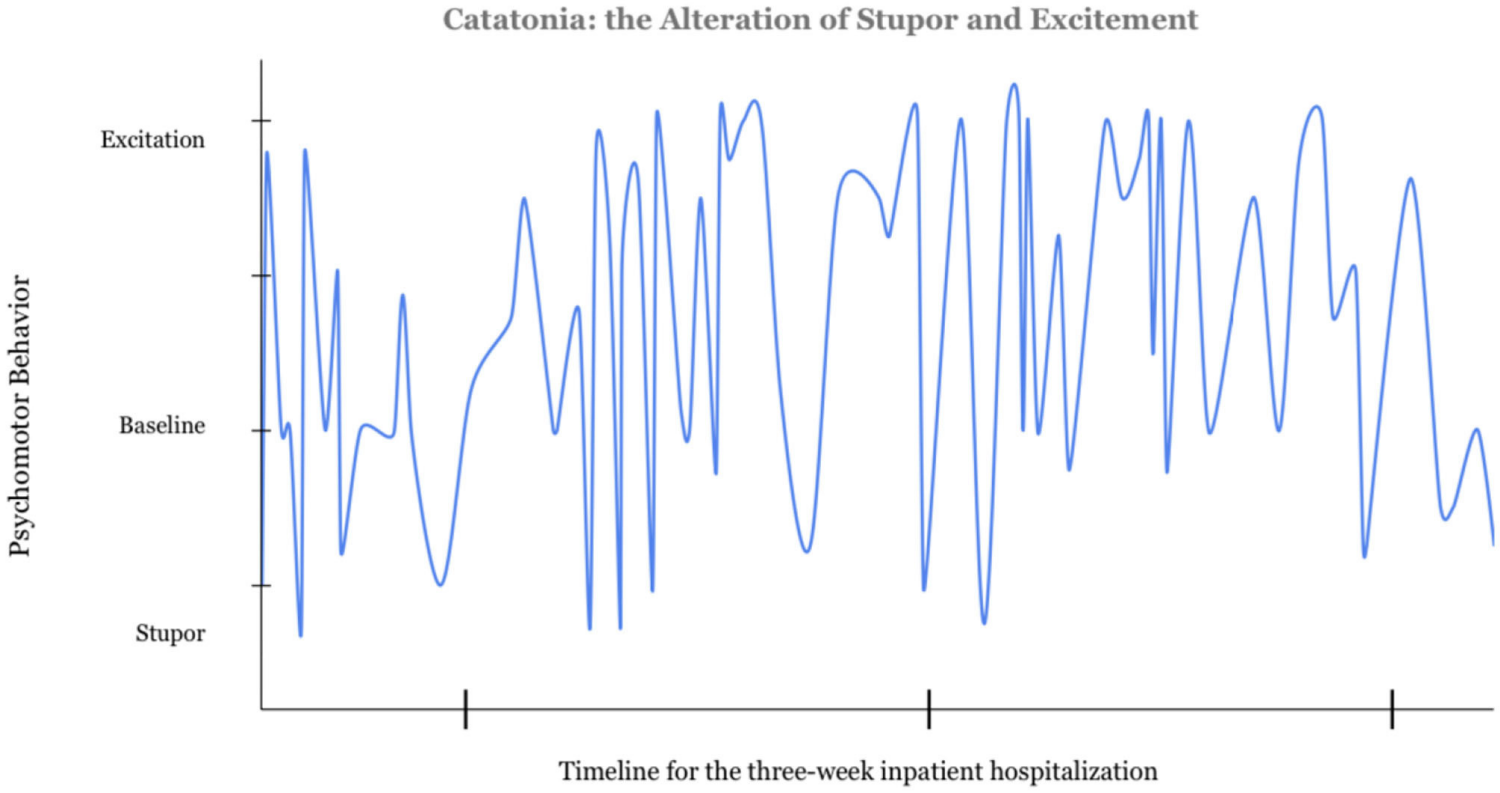
Catatonia: the Alteration in Stupor and Excitation. The data for this graph was gathered from the nursing notes documented over the course of a 3-week admission prior to the diagnosis of catatonia. Four of the authors independently reviewed the notes and rated the psychomotor behavior from −2 to +2. Excitation was scored as a 2, stuporous catatonia was rated a negative 2. Further information on this scale can be found in the Supplementary Material.

Mr. A was prescribed oral mixed amphetamine salts (Adderall) 30 mg daily and risperidone 7 mg daily for the past 7 years by a developmental pediatrician, for ADHD and aggression related to autism. On inpatient psychiatric admission, he exhibited behaviors including intermittent agitation, muttering, and urinating on the floor. He displayed periods of psychomotor agitation as well as stupor (Figure 1). His speech was slow with sporadic muteness, but he had times of clear sensorium, more normal speech, and behavioral control. During an initial brief hospitalization, medication changes included adding sodium valproate (Depakote) 250 mg PO TID for affective stability and impulsivity, lowering risperidone to 3 mg PO BID, and discontinuing mixed amphetamine salts due to potential to worsen insomnia and psychosis.

Mr. A had four subsequent psychiatric admissions of increasing length, and his case perplexed staff and the psychiatric team. He slept 1–2 h per night and was rarely at rest. He displayed purposeless and bizarre stereotypical movements, including repetitive knocking on walls and flapping his arms. He perseverated on activities such as opening and closing his door. At times he muttered or was mute. Staff attributed these behaviors to autism though he had never exhibited such behaviors previously. Disinhibited behavior included episodes of maniacal laughter and compulsive masturbation for up to 5 h. He jumped impulsively into the nurse's station and wandered into other patients' rooms, taking belongings. He flooded bathrooms, gleefully running through the water or urine, yelling “Poseidon!”. He impulsively stabbed a social worker with a plastic fork. Over 3 months and five hospitalizations, he was exposed to more than 10 psychotropic medication including: 3 atypical antipsychotics, 2 first-generation antipsychotics, divalproex, and 4 antihistamines or sedating antidepressants for sleep (Table 1). Diagnoses postulated included: obsessive compulsive disorder (OCD), acute psychotic disorder, schizophreniform disorder, acute stress disorder, delusional disorder, amphetamine-induced psychotic disorder, and cannabis-induced mood disorder.

**Table 1:** Pharmacologic Interventions for each patient.

	**Patient A**	**Patient B**
**Pharmacologic interventions**		
**Dopamine antagonist**		
Aripiprazole	400 mg IM depot	5 mg PO Daily
Olanzapine	15 mg SL BID	2.5 mg PO QHS
Quetiapine	300 mg PO BID	
Risperidone	3 mg PO BID	3 trials, dose unknown
Chlorpromazine	50 mg PO TID	
Haloperidol	5 mg PO TID	
Lurasidone		20 mg PO daily
**Gabanergic medication**		
Clonazepam	1 mg PO TID	
Zolpidem	10 mg PO QHS	
Lorazepam (Max TDD)	22 mg PO and IM	24 mg PO
**Glutamnergic medication**		
Divalproex	500 mg PO BID	
Carbamazepine	200 mg PO BID	
Gabapentin	100 mg PO TID	
**Serotenergic medication**		
Clomipramine	25 mg PO BID	
Sertraline	50 mg QHS	
**Norepinephrine agonist**		
Clonidine		0.3 mg PO daily
**Stimulants**		
Methylphenidate		20 mg PO TID

*The doses listed are the maximum doses prescribed. The agents were often discontinued prior to true “failed trial” due to adverse reactions or worsening of patient's condition*.

Collateral history from his mother indicated clear behavioral change from his baseline unrelated to his autism. A covering psychiatrist, noting an obsession with water, repetitive purposeless behaviors, and unresponsiveness to the external world, made a presumptive diagnosis of catatonia and performed an initial lorazepam challenge of 2 mg intramuscularly (IM) which was markedly positive. Scores following the lorazepam challenge on the Bush Francis Catatonia Rating Scale (BFCRS) decreased from 27 to 12. Mr. A received lorazepam 9 mg IM total that day; repetitive behaviors decreased and speech improved (6). He began following instructions and was oriented to place, time, and situation. On day 5, he received lorazepam 16 mg IM with substantial improvement. The second rotating psychiatrist verified capacity and consent for ECT, tapering lorazepam off in preparation, but Mr. A refused ECT, believing it was a lobotomy. The second psychiatrist believed the patient had progressively worsening symptoms of autism and psychosis, not catatonia. Antipsychotic medication was restarted, and agitation, repetitive behaviors, and aggression toward staff returned. Despite increasing antipsychotic doses over the next 11 days, aggression worsened. On day 18, the relieving psychiatrist stopped all antipsychotics and restarted lorazepam 2 mg IM Q 2 h, with extra doses prn stuporous or repetitive behaviors. Mr. A received 22 mg of lorazepam over 24 h with dramatic improvement in mentation and behavior, and tolerated another 60 mg of lorazepam over the next 80-h period. He was then again oriented, and could clearly articulate his diagnosis. He consented to treatment with his mother present, requested to have ECT immediately to resolve the catatonia and to remove the shooting incident memory, stating, “It's all I have been thinking about.”

Mr. A's first ECT treatment on hospital day 22 resulted in further dramatic response. Lorazepam was held for 10 h until slight stereotypic head turning and muttering returned. The next day he remained at baseline; lorazepam 2 mg po TID was continued. He was discharged home after three additional ECT treatments. He continued outpatient ECT treatments three times per week for a total of 8 treatments, without maintenance ECT, as catatonic symptoms had resolved. He was readmitted within 1 month due to relapse into excited catatonia. Following successful treatment with a short course of ECT, a plan was made for maintenance-ECT (M-ECT) initially every 14 days. Relapses requiring repeat admissions continued for 7 months whenever ECT frequency averaged more than 7 days.

Throughout his admissions, the treatment team debated the diagnosis of catatonia. Mr. A explained his aggressive outbursts and agitation as frustration or anger toward his family. Some staff had not witnessed catatonic behavior due to rapid improvement with ECT. Many attributed less recognizable motor signs of catatonia to his autism, including stereotypies, tics, hand-flapping, finger-snapping, and head-turning.

After 14 months, Mr. A returned to his premorbid functioning, attending school and living with his family, while receiving weekly M-ECT treatments. Attempts to space ECT treatments further apart resulted in recurrent motor symptoms. At the time of this writing, maintenance medication included mixed amphetamine salts XR 30 mg PO daily, olanzapine 20 mg PO daily, sodium valproate 1,000 mg PO BID, and trazodone 50 mg PO QHS. The patient also took cannabidiol for anxiety and sleep.

## Case B

Mr. B, a 17 year-old male with level-3 Autistic Spectrum Disorder (ASD) and accompanying profound intellectual and language impairment, presented to the ED in a state of excited catatonia. Earlier that morning, his mother and his Applied Behavioral Analysis (ABA) therapists (present since childhood) contained him in the bathroom due to uncontrollable agitation and aggression. Mr. B disrobed and crawled out a small window to the backyard, and 911 was called. He did not respond to response team commands and remained frenzied, so was subdued with IM ketamine and transported to a local ED.

Over the previous 8 months, Mr. B had experienced a steady decline in psychomotor functioning, displaying stereotypical purposeless movements including grimacing, posturing, and muscle tensing. He became increasingly negativistic (moving opposite of a request without motive or purpose), and was agitated and aggressive, scratching and biting his mother and the ABA therapists. Engaging in increasingly inappropriate sexual behaviors, he refused to remain clothed and compulsively masturbated. He began restricting his diet and exhibited ritualistic behavior with food. Preoccupied with water, he flooded his home and refused to toilet appropriately. He slept poorly and displayed a widely labile affect with intermittent tearfulness, wailing, and laughing. Medication trials included lurasidone, risperidone, aripiprazole, and olanzapine; all seemed to worsen his condition.

During the deterioration, his child psychiatrist saw him at his ABA day school to assess his safety. His unpredictable behavior prohibited access to primary care and outpatient labs. A dipstick urinalysis on-site was normal. A neurologic exam was remarkable only for hyperreflexia and resistance to passive movement. Mental status exam revealed a non-verbal young man with frenetic purposeless constant movement, appearing distressed, and agitated. Given the suspicion of excited catatonia, his psychiatrist started oral lorazepam, titrating to 6 mg QID, which afforded brief periods of symptom reduction without sedation. Additional oral medications included methylphenidate 30 mg q am and 20 mg q noon, and clonidine 0.2 mg qhs.

In the ED, Mr. B received IV ketamine 500 mg, lorazepam 4 mg, and haloperidol 5 mg over several hours without benefit. Four-point soft restraints were placed for uncontrollable aggression; he flipped in bed wrapping the restraints around his neck. He scored 86 on the Kanner scale (144 maximum) and 53 on the BFCRS (69 maximum). Due to the severity of his condition, the pediatric intensivist coordinated admission to the Pediatric Intensive Care Unit (PICU). Multiple behavioral codes were called for agitation, with six staff members required for physical restraint. The danger posed to Mr. B and staff prompted initiation of continuous IV sedation with dexmedetomidine (DEX).

All four psychiatrists who evaluated him (two board-certified in child and adolescent psychiatry) recommended emergent ECT. The team educated his mother (sole guardian) on risks and benefits of ECT treatment and alternative options; she gave consent. He could not assent due to intellectual disability and mental state.

Multiple interdisciplinary team meetings ensued to review clinical course, diagnosis, and treatment planning. The team anticipated bilateral ECT daily for 5 days to gain the most profound benefit expediently. Remarkable cooperative efforts between hospital administration, nursing staff, and the anesthesia, intensivist, and psychiatric services enabled a first ECT treatment within 30 h of ED presentation. ECT parameters and seizure length are shown in Table 2.

**Table 2:** ECT treatment parameters and seizure duration for each patient.

	**Electroconvulsive therapy treatment parameters and seizure duration**					
**Treatment number**	**Patient A**			**Patient B**		
	**Charge (mC)**	**Seizure duration (seconds)**		**Charge (mC)**	**Seizure duration (seconds)**	
		**Motor**	**EEG**		**Motor**	**EEG**
1	173	119	248	64.108	6.32	6.45
2	115	45	78	234	19	59
3	144	18	54	288	24	25
4	173	29	173	416.512	0 0	19.72
5	144	0	135	576.576	0 0	21 63
6	144	27	55	576	10	30
7	144	42	51	576	27	43
8	173	0	204	576	21	51
	Pulse width: 0.3 ms. Frequency: three treatments weekly Medication: methohexital, succinylcholine, flumazenil, midazolam			Pulse width: 1.0 ms. Frequency: daily treatments for 5 days then every other day Medications: methohexital, succinylcholine, rocuronium, flumazenil		
	First treatment: pre-treated with flumazenil 0.5 mg due to lorazepam administration. The seizure was prolonged despite two doses of midazolam which were likely ineffective because the flumazenil was administered shortly before the stimulus. There were no adverse effects from the prolonged seizure.			Flumazenil 0.5–1 mg was given prior to treatments 1–7 due to benzodiazepine administration. It was continued for the theoretical pro-convulsant benefit. The stimulus was increased for the second treatment because the patient had been placed on midazolam drip 12 mg/h as well.		
Two charge listed indicates two stimulus were required for the treatment. MECTA 5000 mC = millicoulombs						

Due to severe agitation, midazolam 12 mg/h was added to DEX. Given risk for aspiration, he was intubated prior to the second ECT treatment. DEX was weaned due to bradycardia; fentanyl and vecuronium continuous infusions were added for deeper sedation.

As Mr. B had received lorazepam and midazolam within a few hours of the first treatment, flumazenil 0.5 mg was administered prior to the first treatment to reverse their effect of increasing the seizure threshold. Mr. A had required this as well as he was on a high dose of lorazepam prior to initiation of ECT treatments. The flumazenil was continued for each subsequent treatment in Mr. B's series despite the cessation of the midazolam drip prior to treatment five. This was utilized for the theoretical modulation of the gabanergic activity to be pro-convulsant (7, 8).

On day four, prior to ECT#3, sedation and paralytics were tapered off for a thorough neurologic exam. Mr. B was now able to follow simple commands. No focal abnormalities were evident and reflexes were brisk. After ECT#5 on hospital day six, he was extubated; the intensivist gave an 85% chance for reintubation due to aggression and hospital constraints. The dedicated ABA therapy team provided 24-h support post-extubation to help manage his behaviors.

**Figure 2:**
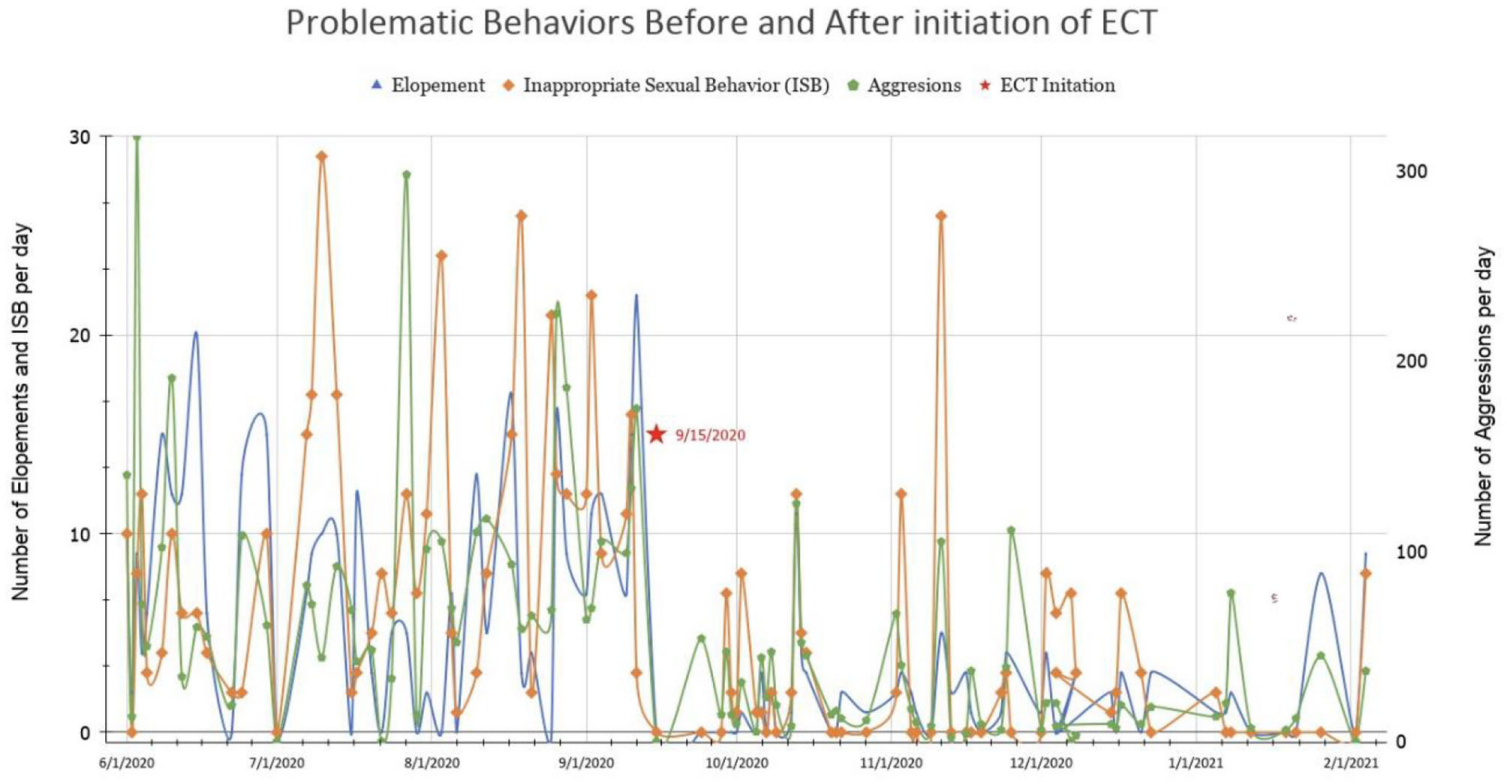
Summary of behavior improvement following ECT initiation for Mr. B.

Mr. B's improvement was more dramatic than expected (Figure 2). Within hours of extubation, he requested dinner and to use a urinal. He tolerated soft bilateral restraints and IV lines. Episodic grimacing and body tensing had resolved. An ABA therapist stated that the functioning seen corresponded to when he was 8 years-old. The PICU nurses were astonished when he said “Thank you, nurse.” He received ECT every other day in the PICU while an outpatient ECT service with adolescent capacity was sought, due to almost certain risk for rapid decompensation without it. Mr. B improved with each treatment. He began to request items spontaneously, answered questions, laughed, and smiled. He no longer required restraints, and sat calmly in bed in street clothes. No further inappropriate sexual behavior occurred. His mother and therapists noted language skills from 4 years prior. He began taking walks without threat of elopement. A tertiary care state university psychiatry program agreed to provide outpatient ECT, and he was discharged home. Mr. B received more than 20 acute series ECT treatments, tapering to weekly treatment. He is significantly improved compared to initial presentation, but with residual symptoms; the burden of weekly regional transport prevents greater frequency of M-ECT and may necessitate residential treatment.

## Discussion

Rates of catatonia on inpatient psychiatric units are estimated at 5–20% (7). Similarly, rates of comorbid catatonia in autism are 12–20% (8). Nevertheless, recognizing catatonic symptoms in those with ASD is challenging: 40 different symptoms of catatonia have been described (9), and the overlap between autism and catatonia creates diagnostic uncertainty (3, 4). Our patients, although both with ASD, had very different levels of baseline functioning and differing courses of excited catatonia. For Mr. A, catatonia was only suspected after five psychiatric admissions despite acute changes in behavior and functioning. Mr. B had a gradual onset of catatonia, possibly over more than 5 years. Mr. A's catatonia was difficult for the psychiatric team to recognize. A full consensus on diagnosis did not happen until the dramatic results from the first ECT treatment. Yet, despite confirmation and continued response to ECT treatments, the diagnosis of excitatory catatonia was repeatedly questioned. He became treatment resistant, as the importance of M-ECT spaced weekly was not initially known by the psychiatrists (10). Concerns were also raised if long-term M-ECT could cause cognitive decline, despite contradictory evidence (11).

In case B, autistic catatonia was confirmed by a positive lorazepam challenge after the escalating aggression was recognized as similar to case A's presentation. This aggression posed high risk of injury to himself and others the longer he remained in an excitatory state (12). The inpatient psychiatric team advocated for emergent ECT daily to obtain fast, effective results.

Most clinicians are familiar with the stuporous presentation of catatonia, including psychomotor retardation and weight loss without overt psychosis. This is a limited view of the syndrome and clearly not what is described above. Both patients suffered from decreased speech, agitation, unprovoked aggression, psychosis, insomnia, urinary incontinence, stereotypic and OCD behaviors, and compulsive masturbation. Both experienced dramatic improvements with ECT and required maintenance treatment.

The majority of catatonic patients demonstrate stupor alternating with excitement (13). Fink identified this alternation as “virtually pathognomonic for catatonia” (13). Psychosis is present in 75% of patients with catatonia; it does not negate the diagnosis of catatonia (14). The combination of comorbid autism, catatonia, and psychosis is called the “Iron Triangle”; psychosis associates more strongly with catatonia in patients with ASD (2). In Kahlbaum's mother tongue, Katatonia — *Spannungs-Totalirresein*, means “tension and total insanity.”(13).

Patients with excited catatonia typically do not recall the agitated state and may confabulate explanations for their behavior (15). Mr. A explained his bizarre behavior as frustration, or anger with his family, when he couldn't recall key details during episodes. This added to diagnostic difficulties as staff, missing suggestibility and inaccuracies, accepted his version as fact, and attributed his behaviors to adolescence and autism.

Education to increase clinician awareness of the need for screening for catatonia should improve recognition and more prompt intervention. About 20 percent of patients with catatonia do not show a positive response to lorazepam challenge, which can also lead to diagnostic delay (16). Such delay may contribute to more difficult-to-treat catatonia; experts emphasize early and rapid intervention with effective means (17–19).

Improved awareness and screening with the Kanner and the Bush-Francis Catatonia Rating Scales upon first presentation of functional decline might have facilitated earlier diagnosis (3, 20).

Once finally diagnosed, both patients faced barriers to continuing appropriate treatment. The team did not know (1) that weekly M-ECT was necessary, or (2) a location for outpatient ECT for adolescents. The first factor resulted in multiple relapses and readmissions to the psychiatric unit for Mr. A. The second delayed effective treatment with ECT for Mr. B as the community lacks pediatric resources. ECT for pediatric patients and inpatient care for low-functioning ASD patients are common obstacles (21). Other barriers for families of ASD youth with dangerous behaviors include how to obtain more intensive services.

Patient B's case poses fascinating neurobiologic questions. The episodes of body tensing and other motor symptoms, present for 14 years and considered self-stimulating behavior, resolved after ECT treatments. Unable to read or write since early childhood, he began to recognize letters and trace them. Fiochone (15) refers to catatonia as an executive function disturbance, begging the question of how Mr. B might have responded if given treatment at an earlier age.

Clinicians should consider catatonia in patients with ASD who display psychomotor changes, paying particular attention to regressed speech and functioning (16, 22, 23). Obtaining collateral information on baseline status and changes in level of functioning (including speech, written communication, aggression, ritualistic behaviors, and toileting), along with performing a catatonia screening tool, can provide crucial information leading to earlier diagnosis and optimal treatment (8). The Kanner and Bush-Francis Rating scales' screening tools are brief, consisting of 10–14 items, facilitating their use in an inpatient setting (6, 20).

## Conclusion

Patients with autism who present to the ED and psychiatric crisis units commonly do so after episodes of agitation and aggression that are unmanageable in the outpatient setting. We report successful diagnosis and treatment of two cases of excited catatonia comorbid with ASD, presenting within a year to a small community hospital. This indicates that catatonia in ASD may be more common than previously recognized. Both suffered delays in diagnosis and treatment, resulting in burdens to the patient, their families and the healthcare system.

Diagnosing catatonia in those with ASD is difficult given the overlaps between catatonic symptoms and behaviors typically seen on the autism spectrum. Screening patients with autism with declines in adaptive functioning with tools such as the Kanner and Bush-Francis Catatonia rating scales may facilitate diagnosis. Clinicians, staff, and caregivers universally struggle with caring for these patients, as they require a different understanding of etiology, more intensive services, greater family involvement, and management of aggression that raises risk for injury. Psychiatric units must increase resources for them. Once diagnosed with catatonia, our patients faced continued barriers to appropriate effective treatments. By improving awareness among the medical community and general public on catatonia and barriers to care, earlier diagnosis and better access to appropriate treatment may occur.

## Data Availability Statement

The raw data supporting the conclusions of this article will be made available by the authors, without undue reservation.

## Ethics Statement

Ethical review and approval was not required for the study on human participants in accordance with the local legislation and institutional requirements. Written informed consent to participate in this study was provided by the participants' legal guardian/next of kin. Written informed consent was obtained from the individual(s), and minor(s)' legal guardian/next of kin, for the publication of any potentially identifiable images or data included in this article.

## Author Contributions

NB, MM, MR, and KG collaborated on the clinical work. NB, MM, and MR completed literature review. BG oversaw the process and contributed to content and editing. All authors contributed to manuscript revision, read, and approved the submitted version.

## Conflict of Interest

The authors declare that the research was conducted in the absence of any commercial or financial relationships that could be construed as a potential conflict of interest.

## Supplementary Material

Supplementary Video 1 The video demonstrates the motor signs of catatonia in a patient with autism spectrum disorder. The overlay of these symptoms with self-stimulating behaviors contributes to the misdiagnosis of the patients with autistic catatonia. This patient only develops these stereotypic movements when experiencing a catatonic episode. The movements resolve with lorazapam electroconvulsive therapy treatments.
