# Supplementary material for: Excited Catatonia in Autism Spectrum Disorder: A Case Series
Source: Front Psychiatry. 2021 May 11;12:674335. doi: 10.3389/fpsyt.2021.674335 (PMC8144290; doi:10.3389/fpsyt.2021.674335)
Supplement: Supplementary file 2 [file Data_Sheet_1.PDF]

Psychomotor Behavior	Description	Rating	Staff Descriptions of Behavior
EXCITED	Constant motion, dangerous activities	2	Running, constant motion, hitting, required restraints or IM medication, danger to staff/self/property, constant pacing, throwing, cartwheels, eloping
AGITATION	increased activity	1	repetitive behaviors, self-harm, intrusive, some pacing
Baseline	calm, cooperative, speaking normally	0	calm, cooperative, oriented, speaking normally, relating information appropriately
Retardation-Withdrawn	decreased activity, isolating	-1	isolative, decreased speech, not eating
CATATONIC	stuporous, mute	-2	Staring, mute, stupor, moving slowly or not at all
